# Soluble RAGE Plasma Levels in Patients with Coronary Artery Disease and Peripheral Artery Disease

**DOI:** 10.1155/2013/584504

**Published:** 2013-10-09

**Authors:** Colomba Falcone, Sara Bozzini, Luigina Guasti, Angela D'Angelo, Anna Clizia Capettini, Edoardo Maria Paganini, Rossana Falcone, Roberto Moia, Carmine Gazzaruso, Gabriele Pelissero

**Affiliations:** ^1^Interdepartmental Center of Research in Molecular Medicine (CIRMC), University of Pavia, 27100 Pavia, Italy; ^2^Department of Cardiology, “Istituti Clinici di Pavia e Vigevano” University Hospital, 27100 Pavia, Italy; ^3^IRCCS San Donato Hospital, San Donato Milanese, 20097 Milan, Italy; ^4^Department of Internal Medicine, IRCCS Policlinico San Matteo, 27100 Pavia, Italy; ^5^Department of Clinical and Experimental Medicine, University of Insubria, 21100 Varese, Italy; ^6^Department of Vascular Surgery, “Istituti Clinici di Pavia e Vigevano”, University Hospital, 27100 Pavia, Italy

## Abstract

The objective of the present study was define in a relatively large patient population with coronary artery disease (CAD) whether the concomitant presence of peripheral artery disease (PAD), which is known to convey additional cardiovascular risk, was associated with different circulating levels of sRAGE with respect to CAD alone and control subjects. Clinical and laboratory parameters including the ankle brachial index (ABI) and sRAGE (enzyme-linked immunosorbent assay kit) were investigated in 544 patients with angiographically documented CAD and 328 control subjects. 213/554 CAD patients (39%) showed an ABI <0.9 associated with typical symptoms (group CAD + PAD), whereas 331 patients were free from PAD. The concentration of plasma sRAGE was significantly lower (P < 0.0001) in CAD population, with and without PAD, than in control subjects. Among CAD patients, those with PAD showed lower levels of sRAGE. The distribution of the three groups (CAD, CAD + PAD, and controls) according to sRAGE tertiles showed that lower levels were more frequent in patients with CAD and CAD + PAD, whereas higher levels were more frequently found in controls. CAD patients presenting with PAD have lower sRAGE levels than CAD patients without peripheral atherosclerosis showing that stable atherosclerotic lesions in different vascular districts are inversely related to soluble decoy receptor sRAGE.

## 1. Introduction

Atherosclerosis is a progressive disease characterized by endothelial disruption, accumulation of lipids, and fibrous elements in arteries [1]. Peripheral artery disease (PAD) is the consequence of the gradual progression of atherosclerosis in the distal aorta, and in the iliac and femoral arteries, eventually leading to stenosis and ischaemia of the lower extremities [2]. In patients with PAD, coronary artery disease (CAD) has a prevalence of 46% to 71% and, in addiction, at least half of patients with CAD have some form of PAD [3, 4]. 

Previous epidemiologic investigations have demonstrated that elevations of plasma markers predict a greater incidence or recurrence of coronary ischemic events or ischemic stroke [5]. Elevations of these factors, associated with inflammatory activity, may reflect the presence of atherosclerotic lesions. Moreover, changes in functional properties of circulating blood cells involved in the atherosclerotic process have been reported [6–11]. Existing data suggest that the relative impact of these exposures in the peripheral vasculature differs from the coronary circulation thereby invoking potential site-specific atherogenic mechanisms [12]. 

In addition to traditional risk factors, new markers appear to have a role in the onset and evolution of both CAD and PAD. In previous studies, lower levels of soluble receptor for advanced glycation end products (sRAGE) were found in hypertensive and CAD nondiabetic patients when compared to healthy subjects [13–15]. RAGE is a member of the immunoglobulin superfamily and is expressed by ECs, SMCs, monocytes, and lymphocytes with enhanced expression in atherosclerotic lesions. The soluble form of RAGE, sRAGE, is a product of both alternative splicing of the gene for RAGE (esRAGE) and cleavage of membrane-bound RAGE [16]. While the biological function of sRAGE has not been clearly defined, one proposed pathological role is that it may act as a competitive inhibitor of ligand-RAGE interaction and the subsequent downstream signaling.

CAD and PAD are expression of the atherosclerotic disease that involves different vascular districts. Based on these observations, we hypothesized that high levels of sRAGE may exert antiatherogenic effects by preventing ligand-triggered RAGE-dependent cellular activation and/or high sRAGE plasma levels may be a marker of antiatherogenic mechanisms acting in the vasculature. Therefore, we sought to find a relationship between sRAGE and the district distribution of atherosclerosis in CAD patients with or without PAD. As a secondary endpoint, we aimed at investigating a potential association between sRAGE concentration and the severity of CAD expressed as the number of vessels showing >50% stenosis in our relatively large patient population affected by documented coronary atherosclerotic lesions.

## 2. Methods

### 2.1. Study Population

The study comprised 544 patients with angiographically documented CAD within the past 6 months before entry into the study and 328 control subjects. Patients were consecutively enrolled at the Cardiology Department of the University Hospital of Pavia and showed at least one coronary stenosis >50% at angiography. Subjects with acute ischemic syndromes, heart failure, or cardiomyopathies were excluded. Exclusion criteria for all study participants also comprised acute infection, acute state of a chronic infections or inflammatory disease, severe liver or renal disease, neoplasm and hematologic disorders, organ transplantation, and pregnancy. 

All patients underwent a detailed clinical interview, physical examination, and standard serum laboratory examination including total cholesterol, triglycerides, high-density lipoprotein (HDL) and low-density lipoprotein (LDL) cholesterol, sRAGE, and high sensitive C-reactive protein (hsCRP). Moreover, a noninvasive vascular assessment of the lower limbs by duplex scanning and ankle and brachial systolic pressure measurement with a Doppler ultrasound probe was performed in all patients. 

In total stable CAD population, there are 125 patients (23%) with previous myocardial infarction and 419 patients (77%) with previous episodes of angina. Of the 544 CAD patients, 317 were on treatment with beta blockers, 290 with angiotensin converting enzyme inhibitors/angiotensin receptor blockers and 263 with statins.

A documented PAD was found in 213/544 (39%) CAD patients. PAD was defined as the presence of an ankle-brachial index (ABI) <0.9. Although for study protocol we chose to exclude asymptomatic subjects and subjects with acute limb ischemia, no subject was asymptomatic and no subject presented acute ischemia. All patients with PAD had typical symptoms, such as claudication or leg pain upon exertion, rest pain, or minor or major tissue loss. Patients with PAD were clinically classified on the basis of the Fontaine classification. For definition of chronic critical lower extremity ischemia, we used AHA guidelines [17]. 

The control subjects were selected from patients who visited our affiliated hospitals or clinics for a physical check-up. Controls were characterized by no history of angina and other heart disease and absence of ischemia induced by maximal exercise stress testing. They matched CAD patients by age, sex, and ethnicity. Controls had no evidence of peripheral artery or cerebrovascular disease and all had normal echo duplex of cervical arteries, the aorta, and lower limbs, with resting and postexercise ankle/brachial pressure index >0.85. 

The severity of CAD was expressed as the number of vessels with >50% stenosis (one-vessel, two vessels, three vessels disease).

Our local ethics committee approved the study protocol. All study participants signed informed consent.

### 2.2. Definition of Cardiovascular Risk Factors

Hypertension was defined by a blood pressure ≥140/90 mmHg or by the use of antihypertensive medications. The diagnoses of diabetes mellitus were inferred in patients already taking oral hypoglycemic therapy or insulin or when fasting plasma glucose >126 mg/dL was found. Cigarettes smoking was dichotomized into ever versus never, with ever smoking defined as having smoked daily for one year at least. The body mass index (BMI) was calculated as weight divided by the square of height. Patients with 18.5 < BMI ≤ 24.9 were considered normal weight, overweight if 25 ≤ BMI ≤ 29.9, obese if BMI ≥ 30. A familiar history of coronary artery disease was identified in relation to the presence of first or second-degree relatives with early coronary events (under 55 years of age for males and under 65 years for females).

### 2.3. Laboratory Methods

In patients and controls, blood samples were taken in EDTA containing tubes after a 14-hour overnight fasting for sRAGE quantification as well as determination of standard laboratory parameters. Blood samples were centrifuged at 1000 g for 30 minutes and immediately divided into aliquots. Plasma specimens were then frozen and stored at −20°C until analysis. 

Plasma sRAGE levels were determined using a commercially available enzyme-linked immunosorbent assay kit (Quantikine; R&D systems) according to the manufacturer's protocol. Briefly, a monoclonal antibody against sRAGE was used to capture sRAGE from plasma. Captured sRAGE was detected with a polyclonal antihuman sRAGE antibody. After washing, plates were incubated with streptavidin-HRP, developed with appropriate substrate, and OD450 was determined using an enzyme-linked immunosorbent assay plate reader. The intra-assay and interassay coefficients of variation values were <6% and <8%, respectively.

Measurements were performed in duplicate and the results were averaged.

### 2.4. Statistical Analysis

The study power was determined by using StatMate 2 for Windows (GraphPad software). 

The Kolmogorov-Smirnov test of normality was used to verify whether the distribution of variables followed a Gaussian pattern. Normally distributed data in groups were expressed as means ± SDs. For continuous variables, the differences between the groups were evaluated with an unpaired *t*-test or ANOVA. Nonparametric variables were expressed as median (interquartile range) and comparisons were done using the Mann-Witney *U* test and Kruskal-Wallis test. Categorical variables are presented by frequency counts, and intergroup comparisons were analyzed by a *χ* ^2^ test. CAD patients and control subjects were categorized into tertiles based on the plasma sRAGE concentration in the entire study cohort. The intertertile cut-off points of plasma sRAGE level were 487 and 932 pg/mL: category 1: <487 pg/mL; category 2: ≥487 pg/mL and <932 pg/mL; category 3: ≥932 pg/mL.

Two-tailed *P* < 0.05 was considered statistically significant.

## 3. Results

### 3.1. Clinical and Laboratory Characteristics of Patients and Control Subjects

The main clinical and biochemical characteristics of the study participants are shown in Table 1. 

CAD cases, with (*N* = 213) and without (*N* = 331) PAD, were more likely to be ever-smokers and hypertensive compared with controls (*P* < 0.0001); in particular, hypertension appears to be more frequent in CAD associated with PAD (77%) with respect to patient with CAD alone (61%) (*P* = 0.0002). In addition, the CAD patients had significantly higher levels of triglycerides compared with controls (*P* < 0.0001). There were no significant differences in age, body mass index, total cholesterol, LDL cholesterol, and HDL cholesterol between the three groups. hsCRP concentrations were higher in CAD patients with respect to control subjects (*P* < 0.01). However, no statistical differences were found between patients with and without PAD.

### 3.2. sRAGE Concentration in CAD Patients with or without PAD and Controls

The concentration of sRAGE in plasma was significantly lower (*P* < 0.0001) in CAD population, with and without PAD, (690 (426–1139) pg/mL) than in control subjects (1335 (936–1954) pg/mL). In particular, among CAD patients, those with PAD also showed lower levels of sRAGE (patients with PAD: 615 (370–1158) pg/mL versus patients without PAD: 766 (474–1226) pg/mL, *P* = 0.02). According to Fontaine classification and our exclusion criteria (see Section 2), the PAD population is composed of 41% of patients in stage IIa, 31% in stage IIb, and 28% in stage III. Similar sRAGE plasma levels were found in patients with stage II and stage III (*P* = 0.11).

**Figure 1:**
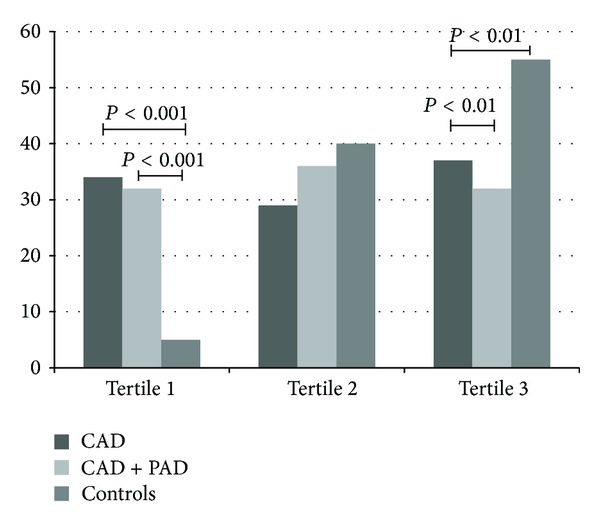
Percentage of subjects with CAD and CAD + PAD and controls according to tertiles of plasma sRAGE concentrations.

Figure 1 shows the percentage distribution of subjects (patients and controls) according to the tertiles of plasma sRAGE concentration. In the first tertile (plasma sRAGE level <487 pg/mL), the number of CAD patients (with and without PAD) was higher than that of the control subjects (*P* < 0.001). Moreover, the number of CAD patients (with and without PAD) in the third tertile (plasma sRAGE level ≥932 pg/mL) was lower than that of the control subjects (*P* < 0.01). There were no statistically significant differences in the prevalence of CAD patients and controls across the second tertile (*P* = 0.25). 

### 3.3. sRAGE Concentration in relation to the Severity of CAD

In relation to the severity of CAD (expressed as the number of coronary vessels affected by atherosclerotic disease), we divided our population of CAD patients into subjects with one, two, or three-vessel disease; sRAGE did not differ among the three groups: one-vessel disease: 578 (217–962) pg/mL; two-vessels disease: 650 (312–1053) pg/mL; three-vessels disease: 692 (336–1150) pg/mL (*P* = 0.4). 

As regard the hsCRP levels, these values did not differ among the three groups of CAD patients with one-vessel, two-vessels, and three-vessels disease (*P* = 0.97). 

### 3.4. Relationship between sRAGE and Laboratory Parameters

Using Spearman's correlation, we examined the association of sRAGE levels with baseline clinical and biochemical features of the study participants. Concentrations of sRAGE were not associated with age, male sex, BMI, blood pressure parameters, diabetes, current smoking or a familial predisposition to CAD or hsCRP (data not shown). 

## 4. Discussion

CAD and PAD are clinical manifestations of atherosclerosis [18, 19]. CAD and cerebrovascular disease are a leading cause of death in adult population when compared to any other medical condition [20] and the presence of PAD has been strongly related to both morbility and mortality [21]. Although the role of immunoinflammatory mechanisms is well known in the pathogenesis of atherosclerosis, the relationship between vascular biomarkers associated with vascular inflammation and the district localization of atherosclerosis (i.e. coronary versus peripheral disease) remains controversial [22]. The main result of this study is the finding of lower sRAGE plasma concentration in CAD patients with PAD with respect to the CAD patients presenting without PAD and to controls. The sRAGE tertiles showed that lower levels were associated with the presence of atherosclerosis (coronary and peripheral), whereas the normal subjects were more likely to be found in the higher sRAGE tertile. 

The mechanisms underlying the finding of decreased sRAGE levels in CAD patients, particularly when PAD was associated with coronary atherosclerosis, remain to be clarified. Interestingly, we reported previously that CAD patients had low circulating sRAGE values even in a subgroup of patients with LDL cholesterol levels of <3.4 mmol/L (130 mg/dL) [13]. In line with the those results, we show here the absence of any relationship between both standard risk factors and hsCRP with sRAGE in patients affected by coronary and coronary plus peripheral obstructive atherosclerotic disease. However, in this study we did not focus on the presence of more complex dysmetabolic clusters such as those found in the metabolic syndrome. sRAGE is a soluble receptor produced by alternative splicing of RAGE mRNA and it is abundantly present in the circulation [23]. Because sRAGE has been shown to successfully bind to AGEs [24], it has been postulated that this soluble isoform could play an antagonistic role by competing with the cell surface receptor, thus preventing the adverse effects of RAGE signaling [25]. sRAGE has been previously administered in several animal models of RAGE-mediated disorders such as diabetic atherosclerosis, altered wound healing, and tumor growth and invasion, in which it successfully prevented or reversed RAGE effects [26–28]. Recently, it has been reported that there is independent, significant and inverse correlation between circulating levels of sRAGE and oxLDL suggesting that part of the antiatherosclerotic effects of sRAGE may be related to oxLDL quenching [29].

The clinical significance of sRAGE plasma levels has been investigated by several studies with controversial results in different ethnicities and in patients with comorbidities such as diabetes and renal failure. Both a positive and negative relationship has been described between sRAGE concentrations and the presence of CAD [13, 30–37]. A recent comprehensive study of 1201 healthy participants in the ARIC Study reported low levels of sRAGE as a marker of future chronic disease since low levels were significantly associated with risk of diabetes, coronary heart disease and mortality [38]. 

In the present study, we did not find a correlation between plasma levels of sRAGE and the severity of CAD with sRAGE in one-vessel disease being statistically similar to the levels found in multivessel disease. It has been reported in patients with non-ST-segment elevation myocardial infarction who developed postpercutaneous coronary intervention restenosis that both low serum sRAGE levels and high AGE/sRAGE ratio were predictive for restenosis [39]. High baseline AGEs but not sRAGE levels were associated with plaque progression investigated by IVUS in the JAPAN-ACS trial [40]. The results of similar sRAGE across the severity of CAD in our patient may be accounted for by the characteristics of this population which included only stable CAD patients. These patients had similar hsCRP among the three groups divided according to the presence of one-vessel, two-vessels and three-vessels disease, suggesting a similar level of systemic inflammation despite the number of vessels diseased. Moreover, in a recent study using a 64-slice computed tomography angiography, the authors showed that circulating sRAGE levels were associated in an inverse manner with plaque burden only in the noncalcified plaque group [33]. Therefore, it has to be acknowledged that our study evaluated CAD by angiography and that other methods investigating the plaque composition may find different results in the relationship between sRAGE levels and severity of CAD.

In our cohort of 544 CAD patients (with and without PAD), concentrations of sRAGE were not associated with age, male sex, BMI, blood pressure parameters, diabetes, current smoking, or a familial predisposition to CAD or hsCRP. Plasma sRAGE levels have been shown to be decreased in diabetic subjects, although conflicting findings have also been reported [32, 41–46]. As regards the potential relationship between traditional serum marker of inflammation and sRAGE, it has been suggested that the synthesis of hs-CRP may be modulated by sRAGE through high levels of TNF-a [47]. Although it is well known that drugs such as statins may reduce cellular and serum marker of inflammation [6–11, 48], the sRAGE may be modulated differently by cardioactive therapies, since it has been reported in the CARDS trial that atorvastatin did not alter sRAGE [33]. Our stable CAD patients were on treatment with vasoactive drugs, which may influence inflammation-related parameters. Indeed, the absolute values of hsCRP in our patient population, though higher in CAD patients than in controls, were relatively low. Therefore, treated patients may not show a significant association between traditional markers of inflammation and sRAGE. Finally, it has to be acknowledged as a limitation of our study that the measurement of total sRAGE cannot discriminate between specific sRAGE splice variants and therefore reduced sRAGE levels measured by this assay may be caused by a reduction of distinct circulating sRAGE isoforms [49]. 

Our present study reports for the first time that CAD patients presenting with PAD have lower sRAGE levels than CAD patients without peripheral atherosclerosis, suggesting that stable atherosclerotic lesions are inversely related to soluble decoy receptor sRAGE and that the measurement of its plasma level may be helpful to evaluate a higher risk of widespread atherosclerosis.

## Figures and Tables

**Table 1:** Clinical characteristics and laboratory parameters of CAD patients with and without PAD and control subjects.

	CAD patients without PAD (*n* = 331)	CAD patients with PAD (*n* = 213)	Control subjects (*n* = 328)	*P* value
Age, y	64.1 ± 5.97	64.5 ± 10.7	63.3 ± 8.3	Ns
Male, *n* (%)	182 (79%)	175 (82%)	272 (83%)	Ns
BMI, kg/m^2^	25.9 ± 2.71	25.7 ± 3.2	25.6 ± 3.1	Ns
Ever-smoking, *n* (%)	232 (70%)**	142 (67%)**	102 (31%)	*P* < 0.0001
Hypertension, *n* (%)	202 (61%)**	164 (77%)**	58 (18%)	*P* < 0.0001
Diabetes, *n* (%)	160 (33%)*	70 (32%)*	33 (10%)	*P* < 0.001
Total cholesterol, mmol/L	5.1 ± 1	4.9 ± 1.16	4.99 ± 1.0	Ns
Triglycerides, mmol/L	3.19 (1.19–12.8)**	2.92 (0.83–9.6)*	1.47 (1.15–2.06)	*P* < 0.0001
HDL cholesterol, mmol/L	1.27 ± 0.36	1.22 ± 0.73	1.40 ± 0.42	Ns
LDL cholesterol, mmol/L	3.17 ± 0.87	3.39 ± 1.23	3.26 ± 1.2	Ns
C reactive protein, mg/dL	1.1 (0.6–1.6)*	1.3 (0.7–1.8)*	0.6 (0.3–1.1)	*P* < 0.01
sRAGE, pg/mL	766 (474–1226)***	615 (370–1158)**	1335 (936–1954)	*P* < 0.0001

*P < 0.05 versus controls; **P ≤ 0.001 versus controls, ***P < 0.05 versus CAD patients with PAD.
